# Wave masking enhances electrocardiogram reconstruction with linear regression

**DOI:** 10.1038/s41598-025-27196-2

**Published:** 2025-12-04

**Authors:** Ekenedirichukwu N. Obianom, Noor Qaqos, Shamsu Idris Abdullahi, G. André Ng, Xin Li

**Affiliations:** 1 Department of Cardiovascular Sciences, University of Leicester, Leicester, UK; 2 School of Engineering, University of Leicester, Leicester, UK; 3 Department of Cardiology, University Hospitals of Leicester NHS Trust, Leicester, UK; 4 Leicester British Heart Foundation Centre of Research Excellence, University of Leicester, Leicester, UK; 5 Leicester National Institute for Health and Care Research Biomedical Research Centre, University of Leicester, Leicester, UK

**Keywords:** ECG, Reconstruction, Correlation, Machine learning, Deep learning, Regression, Biomedical engineering, Data processing, Machine learning

## Abstract

Electrocardiogram (ECG) reconstruction involves synthesizing leads from a reduced or alternative lead set. While ECG leads are generally considered linearly related, recording distortions and individual differences make perfect replication difficult, leading researchers to explore deep learning (DL) methods. This paper challenges DL methods by introducing wave masking, a novel preprocessing technique adapted from image recognition, where sections of the input are masked to highlight segments most relevant to improving reconstruction. Applied to ECG, it emphasizes key parts of the time-series signal. The study compares the performance of wave masking combined with linear regression against traditional preprocessing for both linear and DL models, using 10,000 normal ECG records from the CODE-15% database (trimmed to 10 s, resampled to 500 Hz, and denoised). Results show mean correlation values of 0.869 ± 0.201 for the linear pipeline, 0.880 ± 0.190 for the wave masking pipeline, and 0.894 ± 0.168 for the DL pipeline. Wave masking significantly improves linear regression performance by over 0.01 and produces results comparable to DL models, though not superior. These findings highlight wave masking as a promising, low computation preprocessing step for ECG reconstruction. Further research is needed to explore its potential benefits when integrated with deep learning models and diverse demographic records.

## Introduction

 Electrocardiogram (ECG) is a painless and non-invasive method of measuring the electrical potential of the heart^1^. The standard ECG, which has been used since 1954, is the 12-lead ECG derived from 10 passive electrodes strategically placed around the human body^2^. Four electrodes are placed on the limbs while 6 electrodes are placed on the torso. This standard helps to view the heart from various directions and points of view, allowing physicians to make informed decisions on the diagnosis of the patient whose ECG is being measured.

The standard 12-lead (S12) is composed of leads I, II, III, aVR, aVL, aVF, V1, V2, V3, V4, V5, V6 which are mathematically derived from the 10 electrodes. Despite the versatility of the S12, problems arise during acquisition of the data and the need for compressing the S12 data has fostered the research into alternative methods of obtaining the same information while reducing or changing the number of electrodes being used to acquire it^3–5^. However, these studies face the daunting task of reconstructing the lost, noisy or compressed data.

Reconstruction of ECG leads follow the understanding that all lead signals originate from a uniform region. The differences that occur in the signal shapes are due to the location (inadvertently the body mass of the region) in which the signals are obtained. Lots of methodologies on reconstruction have been generated from this idea^6–11^. Some of these methodologies have used as little as one lead to reconstruct other leads^11^. These methodologies have also explored both linear and non-linear (including artificial intelligence) methods in performing this task.

Due to recent improvements in deep learning (DL) and machine learning (ML) techniques, there is a common notion that non-linear techniques outperform the linear techniques. This assumption is not farfetched as it has been argued that simple linear transforms cannot accurately represent the complexity of the human physiology^12^. Likewise, many researchers have shown that non-linear techniques may be more suited for reconstructing S12^13,14^. However, some researchers have argued otherwise^15,16^.

Regardless of the reconstruction algorithm employed, the importance of ECG reconstruction remains consistent. One key benefit is its ability to synthesise missing or corrupted leads, which is particularly useful in scenarios where signal quality is compromised due to factors such as patient movement or electrode misplacement during recording^10^. Additionally, ECG reconstruction plays a vital role in reducing the number of leads required for recording. Fewer electrodes result in a lower likelihood of noise interference, simplified system circuitry, and reduced setup time. This not only streamlines the recording process but also facilitates quicker diagnosis and clinical response^17^.

In line with this and the possibility that linear techniques could be better suited to ECG reconstruction, this paper aims to introduce an innovative method of improving the results of linear regression methods in reconstruction of ECG called **Wave Masking**. This method focuses on improving the performance of linear regression methods to be as good as or better than non-linear methods.

## Method

### Wave masking

The design of ML systems comprises three major stages which include data pre-processing, model training and model testing. Wave masking is a process within the data pre-processing stage in which the component waves of the data are extracted (by supressing other waves). This technique originates from image recognition methods in DL. It involves obstructing (masking) sections of the input to ensure that emphasis is placed on parts of the input that are relevant to the improvement in the reconstruction of the output^18^. For ECG reconstruction, this involves emphasizing sections of the input time-series signal.

**Fig. 1:**
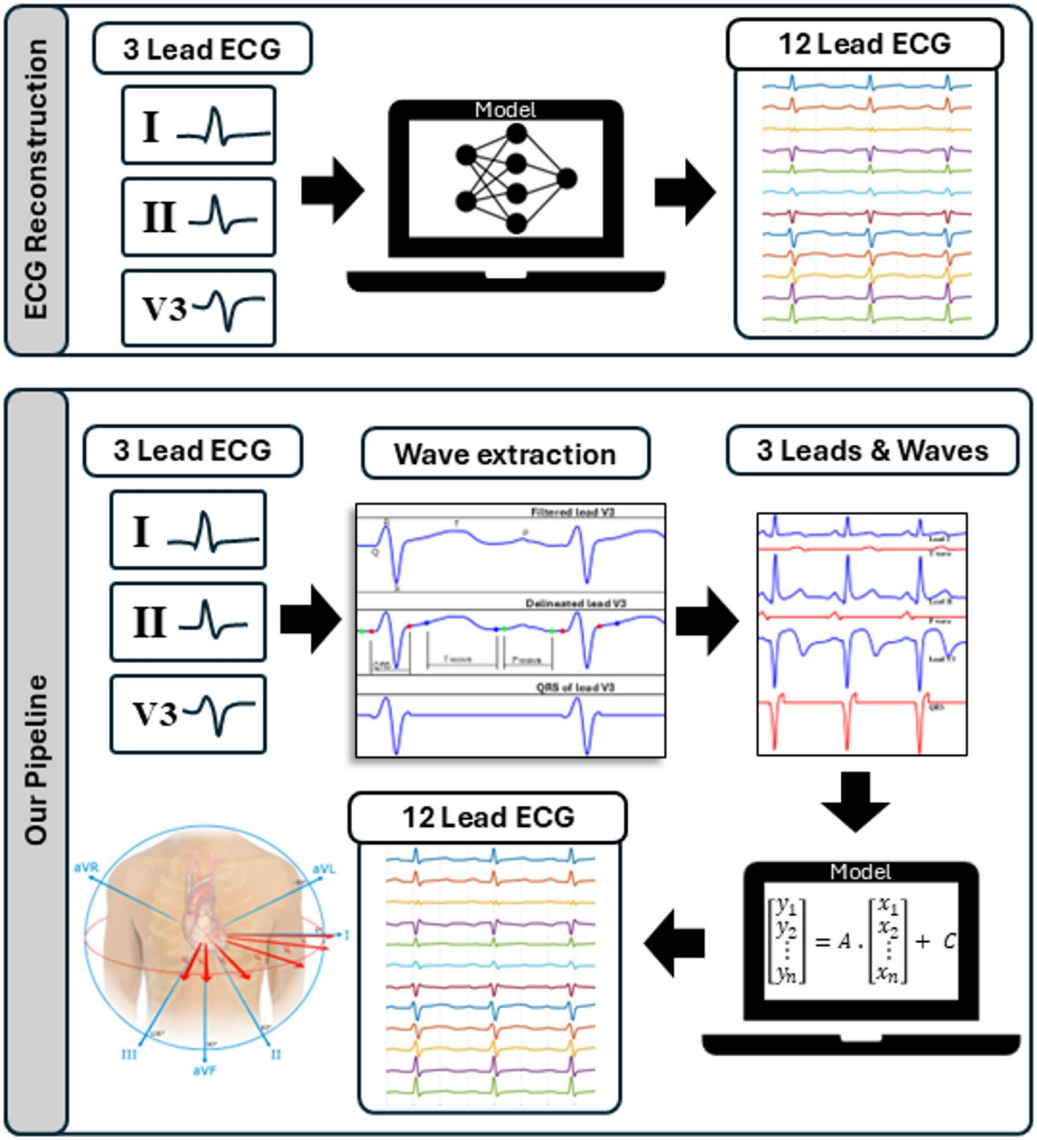
Visualisation comparing the generic ECG reconstruction pipeline to the proposed wave masked pipeline. The proposed pipeline shows the wave extraction process of the QRS complex by zero-masking the T wave and P wave. This procedure is also done to extract the P wave and T wave. The pipeline also shows the new input set (including T wave, P wave, QRS, lead I, lead II, and lead V3) prior to passing it as an input to the model for reconstruction.

ECG signals from healthy patients are made up of periodic waves characterised by certain peaks and troughs^19^. These include P, Q, R, S and T (as shown in Fig. 1) which can be grouped into important segments of the ECG (P wave, T wave and QRS complex) and can be used for wave masking during ECG reconstruction. To extract a particular wave, the signal is first delineated then the sections not containing the wave to be isolated is replaced (masked) with zeros (zero-padding)^20^. Specifically, the P wave can be isolated by masking the T wave and QRS, T wave can be isolated by masking the P wave and QRS, and QRS can be isolated by masking the T wave and P wave. Figure 1 provides a visualization of the process, illustrating the extraction of the QRS complex from lead V3, the T wave from lead I, and the P wave from lead II, prior to combining these components with the original leads for input into the model.

### Dataset

The dataset used here includes 10,000 patients extracted from the CODE-15% dataset^21^. The patients were selected randomly while adhering to the following criteria derived from the metadata:

a. The ECG must not belong to a patient that had been previously selected.

b. The ECG must be categorised as normal.

c. The patient must be false for the ailments monitored during the data collection (which include first degree AV block, right bundle branch block, left bundle branch block, sinus bradycardia, atrial fibrillation, and sinus tachycardia).

d. No lead of the ECG must have a blank signal.

After selection, the signals were trimmed to 10 s, resampled to 500 Hz, denoised, and delineated. The denoising and delineation were performed using a MATLAB toolbox called ECGdeli^22^. It is important to note that the delineation was done using only leads I, II, and V3 to simulate a situation where these are the only available leads during recording. These leads were chosen based on the work done by Butchy, et al^23^..

With respect to leads, Schreck, et al^24^. determined that using three leads as input for reconstruction captured the majority (99.12 ± 0.92%) of the information content in the output. While incorporating more than three leads resulted in a slight improvement, the overall gain was minimal. Since the limb leads (III, aVF, aVL, aVR) can be mathematically derived from I and II, the reconstruction focus was on the precordial leads. Previous attempts on reconstruction have mostly focused on using leads I, II, V2^6,25^to predict V1, V3, V4, V5, and V6. The assumption is that this configuration is often used for reconstruction because of its demonstrated effectiveness without warranting need for the exploration of alternatives leads as input. However, Butchy, et al^23^. performed a lead-to-lead correlation analysis and found that V3 was more correlated with all other leads than V2. The approach in this paper follows the study performed by Butchy, et al^23^. and compares the model performance when using inputs I, II, V3 with respect to using inputs I, II, V2.

### Pipeline design

To analyse the performance of wave masking, five pipelines were designed. These varying designs involved altering the leads used as input, conditional inclusion of wave masking into pre-processing, and altering the model algorithm used for each pipeline. All pipelines were designed to produce five outputs, leads V1, V2 or V3, V4, V5, and V6. Leads III, aVL, aVR, and aVF are easily calculated by mathematical combinations of leads I and II.

#### Pipeline 1 and pipeline 2

**Fig. 2:**
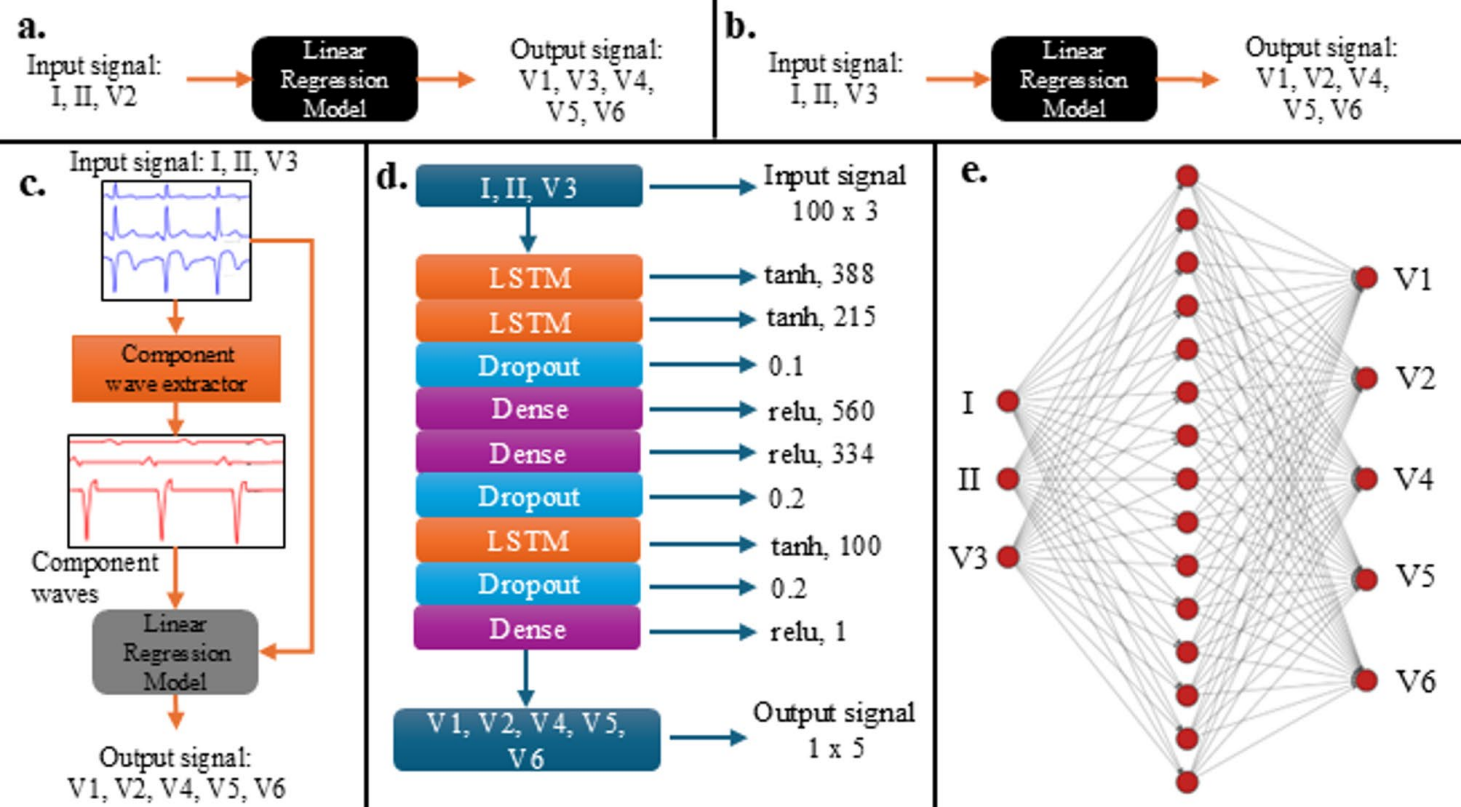
Pipelines - a. Pipeline (1) b. Pipeline (2) c. Pipeline 3 - WMLR. d. Pipeline 4. e. Pipeline 5.

Pipelines 1 and 2 were designed using linear transformation as the model algorithm^6,26,27^. Due to the flexibility of Stochastic Gradient Descent (SGD) in handling large datasets, it was employed to compute the transformation matrix of the linear models. Pipeline 1 used leads I, II, V2 as input (x in Eq. (1)) to the linear regression model (Fig. 2a). Pipeline 2 used leads I, II, V3 as input (x in Eq. (1)) to the linear regression model (Fig. 2b). These pipelines were used to compare the effectiveness of lead V3 over lead V2 in the reconstruction of other leads. The linear transform used in these pipelines are mathematically described by:

$$\:\left[\begin{array}{c}{y}_{1}\\\:\begin{array}{c}{y}_{2}\\\:\vdots\end{array}\\\:{y}_{n}\end{array}\right]=A\:.\:\left[\begin{array}{c}{x}_{1}\\\:\begin{array}{c}{x}_{2}\\\:\vdots\end{array}\\\:{x}_{n}\end{array}\right]+\:C$$

$$\:if,\:y=\left[\begin{array}{c}{y}_{1}\\\:\begin{array}{c}{y}_{2}\\\:\vdots\end{array}\\\:{y}_{n}\end{array}\right]\:and\:x=\left[\begin{array}{c}{x}_{1}\\\:\begin{array}{c}{x}_{2}\\\:\vdots\end{array}\\\:{x}_{n}\end{array}\right]$$

$$\:then,\:y=Ax+C\:\dots\:equation\:\left(1\right)$$

$$\:where:y=the\:potential\:to\:be\:reconstructed$$

$$\:A=matrix\:of\:coefficients$$

$$\:C=column\:vector\:of\:constants$$

$$\:\:x=the\:available\:body\:surface\:potentials$$

#### Pipeline 3: wave masked linear regression (WMLR)

Pipeline 3 (which will be called WMLR: Wave Masked Linear Regression) is very similar to pipeline 2. It computes a linear transformation using SGD. The major difference is that it uses leads I, II, V3, and component waves as input (x in Eq. (1)) to the linear regression model (Fig. 2c). This pipeline is a major step up to see how wave masking affects linear regression.

#### Pipeline 4

Pipeline 4 was designed with long-short term memory (LSTM) as the model algorithm. The LSTM architecture was built with Dhahri, et al^10^. as a guide, however the activation functions of the first 2 layers were changed to tanh to prevent exploding gradient. The model was trained using backpropagation. Pipeline 4 used leads I, II, V3 as input to the LSTM model (Fig. 2d).

#### Pipeline 5

Pipeline 5 was designed with feed-forward (FFN) network as the model algorithm. The FFN architecture was designed with Atoui, et al^9^. as a guide. However, a committee of 5 neural networks was used instead of 50 to reduce training time while benefitting from an ensemble model and an increased dataset. The model was trained using backpropagation. Pipeline 5 used leads I, II, V3 as input to the FFN model (Fig. 2e).

### System requirements

The models were trained and tested using python on ALICE High Performance Computer at the University of Leicester, UK. Pipelines 1, 2, and 3 were trained with a system containing 32 CPUs and 30GB memory. Pipelines 4 and 5 were trained with a system containing 24 CPUs, 1 GPU and 35GB memory.

### Analysis method

A patient-wise 5-fold cross-validation was performed on all pipelines. That is, each pipeline model was trained 5 times on a different 80% of the patients and tested on the remaining 20% of the patients, then the performance was averaged. The performance metrics used to compare these pipelines include the Pearson correlation coefficient and root mean squared error. Correlation is usually used to define the degree to which two signals are linearly related. Its value ranges from − 1 to 1, where 0 means no correlation, −1 means negatively correlated, and 1 means positively correlated.

$$\:r=\frac{\sum\:\left({x}_{j}-\widehat{x}\right)\left({y}_{j}-\widehat{y}\right)}{\sqrt{\sum\:{\left({x}_{j}-\widehat{x}\right)}^{2}\sum\:{\left({y}_{j}-\widehat{y}\right)}^{2}}}$$

$$\:where:\:r=correlation\:coefficient$$

$$\:j=observation\:number\:at\:any\:given\:point\:in\:time$$

$$\:{x}_{j}=original\:sample\:at\:any\:given\:point\:in\:time$$

$$\:\widehat{x}=mean\:of\:all\:original\:values$$

$$\:{y}_{j}=predicted\:sample\:at\:any\:given\:point\:in\:time$$

$$\:\widehat{y}=mean\:of\:all\:predicted\:values$$

Root Mean Squared Error (RMSE) is a metric used to measure the average difference between the predicted model and the actual values. The higher the value, the worse the performance.

$$\:MSE=\frac{1}{N}\sum\:_{j=1}^{N}{\left({y}_{j}-{x}_{j}\right)}^{2}$$

$$\:RMSE=\sqrt{MSE}$$

$$\:where:N=\:total\:number\:of\:observations$$

## Results

### COMPARISM WITH LINEAR ALGORITHMS

WMLR was compared with pipelines 1 and 2. This helped to understand the performance of linear regression model when lead V2 or lead V3 was used as input and the improvements that occurred when component waves were included as inputs. The correlation showed that lead V2 (pipeline 1) performed better in predicting V1 with a lower standard deviation. This is expected, as the proximity between V1 is V2 is closer as compared to V3. Lead V3 (pipeline 2) performed better at predicting other leads except for V2. Including component waves as inputs (WMLR) proved fruitful as it performed better than both pipelines 1 and 2 in predicting all leads.

**Table 1:** Correlation between pipeline 1, pipeline 2 and WMLR.

Predicted	Pipeline 1			Pipeline 2			WMLR		
Leads	Median	Mean	SD	Median	Mean	SD	Median	Mean	SD
V1	0.958	0.896	0.196	0.923	0.839	0.234	**0.922**	**0.843**	**0.227**
V2/V3	0.910	0.81	0.247	0.914	0.808	0.263	**0.920**	**0.825**	**0.244**
V4	0.909	0.827	0.227	0.947	0.887	0.174	**0.956**	**0.895**	**0.171**
V5	0.948	0.899	0.162	0.954	0.909	0.153	**0.961**	**0.918**	**0.144**
V6	0.963	0.913	0.172	0.960	0.913	0.167	**0.964**	**0.918**	**0.163**
Average	0.937	0.869	0.201	0.939	0.871	0.198	**0.945**	**0.880**	**0.190**

**Table 2:** RMSE between pipeline 1, pipeline 2 and WMLR.

Predicted	Pipeline 1			Pipeline 2			WMLR		
Leads	Median	Mean	SD	Median	Mean	SD	Median	Mean	SD
V1	0.082	0.118	0.194	0.107	0.146	0.193	**0.105**	**0.144**	**0.187**
V2/V3	0.191	0.238	0.213	0.178	0.221	0.214	**0.166**	**0.210**	**0.212**
V4	0.190	0.238	0.219	0.145	0.192	0.210	**0.133**	**0.183**	**0.205**
V5	0.144	0.187	0.205	0.134	0.179	0.203	**0.127**	**0.170**	**0.193**
V6	0.107	0.156	0.240	0.107	0.156	0.239	**0.102**	**0.151**	**0.233**
Average	0.143	0.187	0.214	0.134	0.179	0.212	**0.127**	**0.171**	**0.206**

**Fig. 3:**
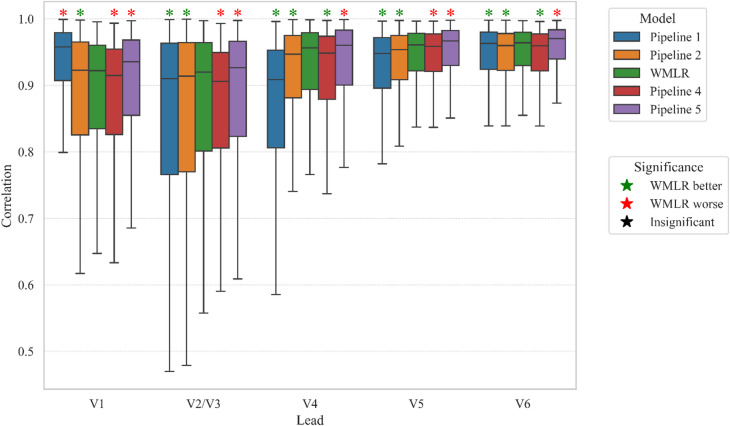
Box and whiskers diagram of the correlation spread of each of the models across the 10,000 tested patients. The box represents the interquartile range, the line inside the box indicates the median, and the whiskers denote the minimum and maximum values. The significance (*p* < 0.05) of each predicted lead of each pipeline against the WMLR based on t-test is shown with a star above each box and whisker. The red star means WMLR was significantly worse, the green mean significantly better, and the black means no significant difference.

The RMSE also shows a very similar performance of the 3 pipelines (Table 2). V2 was superior at predicting V1. Pipelines 1 and 2 performed similarly when predicting V6. WMLR has an averagely better performance. These results can also be visualised from Fig. 3, where the medians, interquatile range and minimum and maximum values are plotted. From this figure it is also clear that the interquatile range of the correlation on all the leads except V1 reduced with WMLR. Paired t-tests were also performed on the RMSE results. P-values less than 0.05 as a threshold to consider significant differences. There was significant difference between pipeline 1 and WMLR in predicting V1. This is consistent with what was viewed in Tables 1 and 2. Pipeline 1 is significantly better at predicting V1. Pipeline 2 is significantly better than pipeline 1 in predicting leads V2/V3, V4, and V5. WMLR is significantly better than pipeline 1 and 2 in predicting all leads (except lead V1, where pipeline 1 is better).

### Comparism with non-linear algorithms

WMLR was also compared with pipelines 4 and 5. This helped to understand how the improvements to using linear regression algorithms compares to the expected superior performance of non-linear algorithms. The correlation showed that WMLR performed better than pipeline 4 in predicting some leads. The mean and median produce very different perspectives of which lead pipeline performs better of the two. However, it can be seen from Fig. 3 that the interquartile range also supports the WMLR as the better option for all leads except lead V2. Pipeline 5, on the other hand, outperformed better than WMLR on all leads. The RMSE shows a similar trend to the correlation results.

**Table 3:** Correlation between WMLR, pipeline 4 and pipeline 5.

Predicted	WMLR			Pipeline 4 Dhahri, et al^10^.			Pipeline 5 Atoui, et al^9^.		
Leads	Median	Mean	SD	Median	Mean	SD	Median	Mean	SD
V1	**0.922**	**0.843**	**0.227**	0.915	0.849	0.191	0.935	0.867	0.195
V2	**0.920**	**0.825**	**0.244**	0.906	0.833	0.199	0.927	0.847	0.209
V4	**0.956**	**0.895**	**0.171**	0.948	0.885	0.171	0.960	0.903	0.161
V5	**0.961**	**0.918**	**0.144**	0.959	0.924	0.119	0.967	0.928	0.128
V6	**0.964**	**0.918**	**0.163**	0.960	0.916	0.145	0.970	0.928	0.148
Average	**0.945**	**0.880**	**0.190**	0.937	0.881	0.165	0.952	0.895	0.168

**Table 4:** RMSE between WMLR, pipeline 4 and pipeline 5.

Predicted	WMLR			Pipeline 4 Dhahri, et al^10^.			Pipeline 5 Atoui, et al^9^.		
Leads	Median	Mean	SD	Median	Mean	SD	Median	Mean	SD
V1	**0.105**	**0.144**	**0.187**	0.112	0.147	0.181	0.099	0.134	0.182
V2	**0.166**	**0.210**	**0.212**	0.169	0.207	0.194	0.152	0.192	0.196
V4	**0.133**	**0.183**	**0.205**	0.146	0.191	0.198	0.125	0.171	0.197
V5	**0.127**	**0.170**	**0.193**	0.131	0.167	0.176	0.121	0.159	0.180
V6	**0.102**	**0.151**	**0.233**	0.113	0.156	0.224	0.096	0.141	0.224
Average	**0.127**	**0.171**	**0.206**	0.134	0.173	0.195	0.119	0.159	0.196

The correlation can be visualised from Fig. 3, where the medians, interquatile range and minimum and maximum values are plotted. It can be seen that due to the distribution of the results, the medians are well above the 0.9 correlation. Paired t-tests were also performed on the RMSE values. All p-values were below 0.05. This signifies that there was significant difference between WMLR and the other pipelines. This means that WMLR performed significantly better than pipeline 4 on leads V4, and V6, but performed significantly worse than pipeline 5 on all leads.

## Discussion

WMLR is grounded in the understanding that improvements in machine learning output accuracy do not always stem from changes to the model architecture. Instead, significant enhancements can be achieved by refining the preprocessing steps. This is especially important as its preprocessing technique (wave masking) is not computationally intensive and can be implemented with minimal additional hardware requirements. This makes it well-suited for integration into existing systems. Moreso, the performance of WMLR may differ statistically from that of deep learning models, its results remain comparable (Fig. 3) and offer a viable alternative, particularly in scenarios where computational resources are limited. Nonetheless, further evaluation is needed, particularly through expert review, to determine whether clinicians would arrive at the same diagnoses when presented with results from WMLR compared to those from deep learning models.

### Analysis against linear model pipelines

The comparison of pipeline 1 and 2 supports the findings of Butchy, et al.^23^, which proposes the use of limb leads and V3 for reconstruction. However, the performance of pipeline 1 in reconstructing V1 is expected, as the V2 recording is in closer proximity to V1 compared to V3. Recognising the relevance of pipeline 2, WMLR was introduced to further improve the performance of linear regression models.

**Fig. 4:**
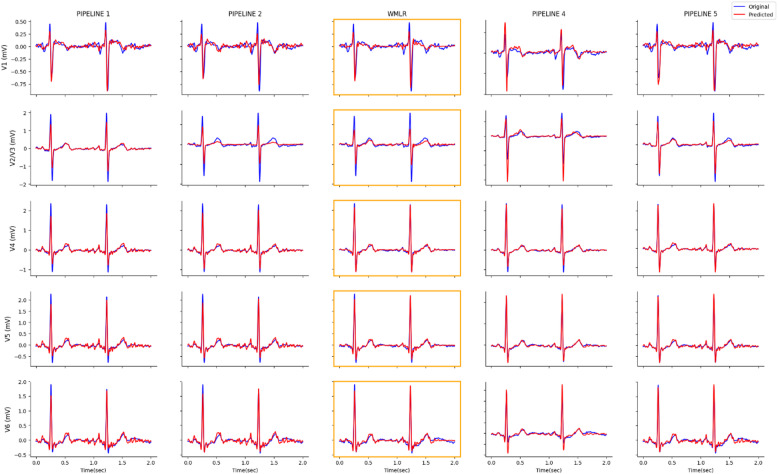
Comparative Diagram of a healthy patient’s ECG reconstructed with the different Pipelines.

In WMLR (pipeline 3), incorporating wave masking step during preprocessing led to measurable enhancements in linear model performance. The process not only showed a significant reduction in RMSE but also demonstrated higher correlation when compared to the standard preprocessing steps when reconstructing using linear regression (Figs. 3 and 4). Although pipeline 1 is still significantly better at reconstructing V1, there is significant improvement in the reconstruction of other leads.

### Analysis against Non-linear model pipelines

It is also relevant to test the performance of WMLR against DL models as the improvements there promise better results over traditional models^13,14^. WMLR was compared against pipeline 4 (a replica of Dhahri, et al^10^.) and 5 (a replica of Atoui, et al^9^.). The average values presented in Tables 3 and 4 indicate some difference in performance among the three pipelines, with WMLR outperforming pipeline 4 based on median but pipeline 5 outperforming WMLR consistently. The paired t-test reveal that WMLR is significantly different to the two pipelines across all leads (Figs. 3 and 4). This means that though pipeline 5 is significantly better than WMLR, WMLR can still demonstrates comparable performance to DL as it is significantly better than pipeline 4 in leads V1, V4, and V6 based on mean correlation. A simple indication to the comparable performance is evident in the visual similarity of the synthesised leads and the originals as shown in Fig. 4. In clinical practice, morphological accuracy is of primary importance to clinicians^28^, and this requirement is satisfied. In addition, WMLR offers a significant advantage in terms of speed, requiring only a fraction of the time needed by DL methods, making it more valuable in medical emergencies.

The implications of this are massive as it proves that complex DL models do not need to be built for reconstruction of ECG. An augmentation of the preprocessing step could be better than designing computing intensive models. Wave masking requires less computing power as compared with DL models. It is a low-level computing process of delineation and extraction of component waves. This also means that models can be developed more efficiently, using lesser resources and enabling faster deployment for real-time reconstruction.

### Clinical significance

Firstly, wave masking for ECG reconstruction supports the growing notion that synthetic leads can serve as clinically reliable substitutes for directly recorded leads. By enabling accurate reconstruction, this approach paves the way for reduced-lead ECG devices that eliminate the need for six additional electrodes during recording. While the cost of individual electrodes and patches may appear minimal, the impact becomes significant at scale. For example, under a traditional setup, 20 sets of electrodes would be sufficient for only two patients (10 electrodes per patient). In contrast, with a reduced lead system requiring only four electrodes per patient, those same 20 sets could serve five patients effectively reducing electrode usage costs by a factor of 2.5.

Moreover, this approach facilitates the development of simpler and more cost-effective embedded systems. With fewer hardware components required, greater emphasis can be placed on software design, enhancing flexibility, maintainability, and integration in portable and wearable medical devices. Furthermore, the wave masking process is not computationally intensive, making it well-suited for integration into existing device architectures. Its lightweight nature allows for seamless implementation without imposing significant demands on processing power or memory, enabling efficient adoption even in systems with limited computational capacity.

In emergency situations, the use of a reduced lead set and less computationally intensive system architecture becomes particularly advantageous. First responders benefit from faster setup times, and the system can synthesise the remaining leads more quickly, all while utilising a device that is both simpler and more cost-effective. Moreso, the low computational demand of the reconstruction process opens the door to the development of more affordable wearable devices. This would significantly enhance the accessibility of remote patient monitoring, enabling physicians to concentrate their attention on patients who require in-hospital care, while those with less critical conditions can be safely and effectively monitored from home^29^.

## Limitations and future work

Wave masking remains in the early stages of development, and there are acknowledged limitations in the current work. Preliminary studies in this area^16,20^ yielded slightly different results, which may be attributed to variations in the datasets used, including differences in the number of patients, demographic characteristics such as race, and the presence of varying cardiac conditions and abnormal heart rhythms. These discrepancies highlight the need for further investigation to ensure the consistency and generalisability of wave masking across different populations, diverse cardiac conditions and clinical scenarios. The preliminary studies also reported high reconstruction performance when abnormal heart rhythms were used as input, which is an encouraging result but one that requires deeper exploration in future work to understand its robustness across varying conditions. In relation to noisy channels during ECG reconstruction, previous work have demonstrated that linear transforms can still yield promising results in such scenarios^30^. Although performance declines rapidly as noise increases, this aspect should be further examined with WMLR, as it enhances reconstruction accuracy and may mitigate the rate of performance degradation under noisy conditions. As previously noted, additional evaluation is also necessary to determine whether clinicians would arrive at the same diagnoses when interpreting results generated by WMLR compared to those produced by other established models. Such validation is essential to assess the clinical reliability and practical applicability of this approach.

Further research into wave masking could enhance the masking process by exploring alternative padding techniques and identifying the most suitable leads for extracting specific ECG waveforms. Such enhancements have the potential to improve model accuracy by introducing only minimal modifications to the preprocessing stage. Additionally, wave masking is not limited to linear regression models; it holds promise as a general preprocessing technique applicable across a variety of modelling approaches. Exploring its integration with DL models and evaluating how these extracted signal features influence model performance would be a valuable direction for future research. This could provide deeper insights into the adaptability and effectiveness of wave masking across different algorithmic frameworks.

## Conclusion

Wave masking is a new technique in ECG reconstruction preprocessing. It involves the delineation and extraction of desired waves that can be used as additional inputs for better reconstruction of ECG signals. This method significantly improved the performance of linear regression models, as can be seen from the comparative results between WMLR and pipelines 1 and 2. It has also improved linear regression models to be comparable in performance to DL models when building generic reconstruction models.

The application of wave masking is limitless as it is a preprocessing step. It has proven useful in ECG reconstruction but can be used in many other tasks. It is relevant in any domain where monitoring of a particular wave or section of a signal needs to be focused on. It also proposes the reduction in complexity of models because it has proven that simple models could produce better results by improving or changing the preprocessing step.

It is also important to acknowledge that wave masking is still in its early stage of development. There is significant potential for further advancements to enhance its effectiveness as a preprocessing step beyond ECG reconstruction. One key area for improvement is the choice of delineation algorithm. According to this Obianom, et al.^20^, this work used Pilia, et al.^22^ for delineation, but alternative approaches may yield better results. Similarly, the padding technique used in wave masking warrants further investigation, as identifying the optimal padding method could enhance its performance. Overall, wave masking is an emerging preprocessing technique with the potential to revolutionize ECG reconstruction and facilitate the adoption of reduced lead systems for medical diagnosis.

## Data Availability

Data used in this work is called “CODE-15%: a large-scale annotated dataset of 12-lead ECGs” and can be accessed with this link: [https://zenodo.org/records/4916206](https:/zenodo.org/records/4916206). Code used for analysis is available based on request [eno3@leicester.ac.uk](mailto: eno3@leicester.ac.uk) or [xin.li@leicester.ac.uk](mailto: xin.li@leicester.ac.uk).
